# Association between use of oral hypoglycemic agents in Japanese patients with type 2 diabetes mellitus and risk of depression: A retrospective cohort study

**DOI:** 10.1002/prp2.536

**Published:** 2019-11-21

**Authors:** Hayato Akimoto, Kotoe Tezuka, Yayoi Nishida, Tomohiro Nakayama, Yasuo Takahashi, Satoshi Asai

**Affiliations:** ^1^ Department of Biomedical Sciences Nihon University School of Medicine Tokyo Japan; ^2^ Clinical Trials Research Center Nihon University School of Medicine Tokyo Japan; ^3^ Department of Pathology and Microbiology Nihon University School of Medicine Tokyo Japan

**Keywords:** depression, dipeptidyl peptidase‐4 inhibitor, oral hypoglycemic agent, type 2 diabetes mellitus

## Abstract

Type 2 diabetes mellitus (T2DM) is a risk factor for depression. Since brain insulin resistance plays a potential role in depression, the future risk of depression in patients with T2DM may be altered depending on the class of oral hypoglycemic agent (OHA) used for T2DM therapy. The aim of the present study was to determine if specific classes of OHAs are associated with a risk for comorbid depression in T2DM. Japanese adult patients with T2DM (n = 40 214) were divided into a case group (with depression; n = 1979) and control group (without depression; n = 38 235). After adjustment for age [adjusted odds ratio (AOR) for 10 years: 1.03; 95% confidence interval (CI): 0.99‐1.07; *P* = .1211], sex [AOR for female: 1.39; 95% CI: 1.26‐1.53; *P* < .0001], hemoglobin A_1c_ [AOR for 1.0%: 1.18; 95% CI: 1.11‐1.26; *P* < .0001], duration of T2DM [AOR for 1 year: 1.00; 95% CI: 0.99‐1.01; *P* = .4089], and history of seven medical conditions, the odds ratios for the development of depression was significantly lower for dipeptidyl peptidase‐4 (DPP‐4) inhibitors [AOR: 0.31; 95% CI: 0.24‐0.42; *P* < .0001]. However, there was no significant association for the other classes of OHAs. Therefore, this study finds that there is less risk of depression associated with the use of DPP‐4 inhibitors for the treatment of T2DM.

**Abbreviations**
AOR: adjusted odds ratio
BBB: blood‐brain barrier
CDW: Clinical Data Warehouse
DPP‐4: dipeptidyl peptidase‐4
FDR: false discovery rate
GLP‐1: glucagon‐like peptide‐1
HbA_1c_: hemoglobin A_1c_
ICD‐10: International Classification of Diseases
NGSP: National Glycohemoglobin Standardization Program
NUSM: Nihon University School of Medicine
OHA: oral hypoglycemic agent
SGLT‐2: sodium glucose cotransporter‐2
STAR*D: sequenced treatment alternatives to relieve depression
T2DM: type 2 diabetes mellitus

## 1 INTRODUCTION

The worldwide prevalence of depression and depressive symptoms has been increasing in recent decades,^1^ and the number of patients with depression globally has reached 300 million.^2^ Depression accounts for the biggest share of the world's burden of disease.^3^

The number of patients with type 2 diabetes mellitus (T2DM) is increasing, and there will be 380 million people with T2DM by 2025.^4^, ^5^ In order to treat T2DM, the use of oral hypoglycemic agents (OHAs), including metformin, α‐glucosidase inhibitors, sulfonylureas, thiazolidinediones, dipeptidyl peptidase‐4 (DPP‐4) inhibitors, and sodium glucose cotransporter‐2 (SGLT‐2) inhibitors, is recommended.^6^, ^7^ In particular, metformin (the first choice in T2DM) and incretin‐related drugs such as DPP‐4 inhibitors and glucagon‐like peptide‐1 (GLP‐1) analogs have good glycemic control.^8^, ^9^, ^10^, ^11^

Recently, an association between T2DM and depression has been reported in several studies with a cross‐sectional design.^12^, ^13^, ^14^ The prevalence of depression in patients with T2DM tend to be low in East Asia compared to Europe and the US (China 6.1%; UK 9.3%; US 10.6%; Spain 32.7%). Additionally, in each country, the prevalence of depression in female patients is larger than male patients.^14^, ^15^, ^16^, ^17^ In addition, it has been suggested that T2DM is one of the risk factors for depression.^18^, ^19^ Furthermore, it is known that DM and depression are independent risk factors for dementia.^20^, ^21^ It is suggested that DM is associated with brain atrophy, particularly hippocampal atrophy in a study using brain MRI.^22^ Patients with comorbid diabetes mellitus and depression have increased risk of dementia.^23^ Therefore, it is important to prevent the development of depression in patients with T2DM.

It is reported that mouse models of high‐fat diet and T2DM exhibit not only depressive‐like behavior but also insulin resistance in the brain.^24^, ^25^, ^26^, ^27^ In mice with brain‐specific knockout of the insulin receptor, brain insulin resistance induces decreased dopamine turnover, leading to anxiety and depressive‐like behaviors.^28^ In human, it has been conceived that peripheral insulin resistance metastasizes to the brain.^29^ Peripheral insulin resistance causes hyperinsulinemia, which enhances lipolysis. Enhanced lipolysis release proinflammatory cytokine and produce reactive oxygen species (ROS). As a result, neuroinflammation and brain insulin resistance occurs. Thus, improvement of brain insulin resistance may play an important role in the prevention and treatment of depression.^29^, ^30^

The risk of depression may be altered depending on the class of OHAs used for T2DM therapy. Hence, the aim of the present study was to characterize the development of depression in T2DM patients being treated with different classes of OHAs.

## 2 METHODS

### 2.1 Data source

The present study was a retrospective cohort study using a clinical database, the Nihon University School of Medicine (NUSM) Clinical Data Warehouse (CDW). NUSM CDW is a centralized data repository that integrates separate databases, including patient demographics, diagnosis, and laboratory data, from the hospital information systems at three hospitals affiliated with NUSM; Nihon University Itabashi Hospital (“I”), Nerima Hikarigaoka Hospital (“H”), and Surugadai Nihon University Hospital (“S”). To protect patient privacy, patient identifiers are replaced by anonymous identifiers in all databases of the CDW.

### 2.2 Study population

Patients aged 20 and older who had been diagnosed with T2DM for at least 30 days were extracted from NUSM CDW (between 2004 and 2018). These patients included patients treated with OHA monotherapy and patients treated with a combination with OHAs, as well as patients with T2DM who had not taken any OHAs (treated with diet and exercise, “non‐use” in Table 1). DM was diagnosed according to the Committee for the Classification and Diagnosis of Diabetes Mellitus of the Japan Diabetes Society (defined as fasting plasma glucose level ≥126 mg/dL, casual plasma glucose level ≥200 mg/dL, plasma glucose 2 h after 75 g glucose load ≥200 mg/dL, or hemoglobin A_1c_ (HbA_1c_, NGSP) level ≥6.5%).^31^

**Table 1:** Characteristics of T2DM patients with and without depression

Characteristics	T2DM patients (n = 40,214)		FDR‐adjusted *P* value
	With depression (n = 1,979, case group)	Without depression (n = 38 235, control group)	
Age (years), mean ± SD	60.48 ± 13.71	60.69 ± 13.36	.5247
Female, n (%)	1020 (50.54%)	15 539 (40.64%)	<.0001
HbA_1c_ (%), median (IQR)	5.77 (0.79)	5.97 (1.18)	<.0001
Duration of T2DM (years), median (IQR)	3.64 (5.82)	3.05 (5.82)	<.0001
Hospital			.0504
Surugadai	477	10 175	
Itabashi	1192	22 173	
Hikarigaoka	310	5887	
Medical history, n			
Arrhythmia	1179	19 713	<.0001
Hyperlipidemia	1283	21 554	<.0001
Hypertension	1244	21 570	<.0001
Rheumatoid arthritis	383	4980	<.0001
Thyroid disease	1028	14 405	<.0001
Liver disease	1285	20 576	<.0001
Kidney disease	729	13 879	.6278
Oral hypoglycemic agents, n			
Nonuse (diet and exercise)	1756	29 868	<.0001
Sulfonylureas	112	3404	<.0001
α‐glucosidase inhibitors	82	2114	.0096
DPP‐4 inhibitors	54	3731	<.0001
Biguanides	41	1905	<.0001
Thiazolidinediones	29	1072	.0005
Glinides	21	751	.0054
SGLT‐2 inhibitors	1	378	<.0001

Note

Student's *t*‐test was performed for differences in age (skewness = −0.63, kurtosis = −0.09 in case group, and skewness = −0.49, kurtosis = −0.08 in control group). Wilcoxon rank‐sum test was performed for differences in HbA_1c_ (skewness = 2.17, kurtosis = 8.29 in case group, and skewness = 1.78, kurtosis = 4.82 in control group) and duration of T2DM (skewness = 1.65, kurtosis = 3.91 in case group, and skewness = 1.79, kurtosis = 4.27 in control group). Chi‐squared test was performed for differences in categorical variables.

Abbreviations: DPP‐4, dipeptidyl peptidase‐4; FDR, false discovery rate; IQR, interquartile range; SD, standard deviation; SGLT‐2, sodium glucose cotransporter‐2; T2DM, type 2 diabetes mellitus.

The experimental protocol was approved by the Ethics Committee of the NUSM (approved number: 31‐9‐0), and the study was conducted in compliance with the Ethical Guidelines for Medical and Health Research Involving Human Subjects of the Ministry of Education, Culture, Sports, Science and Technology and the Ministry of Health, Labour and Welfare, Japan.

### 2.3 Neuropsychiatric evaluation

In the present study, the presence or absence of development of depression was regarded as the outcome. The diagnosis of depression was made by medical doctors belonging to either of these three hospitals (according to the International Classification of Diseases [ICD‐10] code; F320, F328, F329). Patients with T2DM were divided into “case group (depression)” and “control group (non‐depression)” by the presence or absence of depression. Furthermore, to avoid reverse causation bias, patients who had developed T2DM after the onset of depression were excluded.

It is known that the use of antipsychotic drugs such as olanzapine and quetiapine is a risk factor for hyperglycemia.^32^, ^33^ In addition, Alzheimer's disease (ICD‐10 code; G30.0 ‐ G30.9) and vascular dementia (F01.0‐F01.9) are associated with depression.^34^, ^35^ Therefore, patients who had used these antipsychotic drugs before the onset date of depression, and patients with a history of these types of dementia were excluded from analysis.

### 2.4 Co‐variables to assess the risk of depression

Age, sex, hospital (I, H, and S), duration of T2DM, HbA_1c_, and medical history were considered as potential confounding factors in this study.

**Figure 1:**
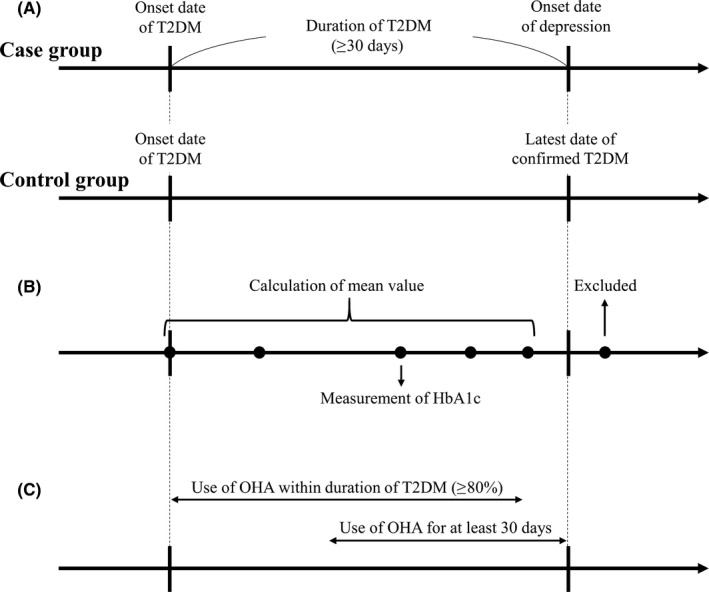
Definition of duration of T2DM (A), glycemic control (B), and use or nonuse of oral hypoglycemic agents (C). T2DM, type 2 diabetes mellitus; HbA_1c_, hemoglobin A_1c_; OHA, oral hypoglycemic agent

The duration of T2DM in patients of the case group was defined as the number of days from the onset date of T2DM to the onset date of depression (Figure 1A). Furthermore, the duration of T2DM in patients of the control group was defined as the number of days from the onset date of T2DM to the latest date of diagnosis of T2DM. The duration of T2DM was at least 30 days.

As the degree of glycemic control is associated with symptoms of depression as well as some type 2 diabetes‐related complications,^36^, ^37^ glycemic control may be a potential risk factor for the development of depression. Therefore, mean value of HbA_1c_ measured at least two times before the onset date of depression was calculated as an index of glycemic control (Figure 1B).

Medical history included information on arrhythmia (ICD‐10 code; I42‐I50), hyperlipidemia (E78.0‐E78.5), hypertension (I10), rheumatoid arthritis (M05, M06, and M08), thyroid disease (E00‐E07), liver disease (K70‐K77), and kidney disease (N00‐N19) diagnosed before the onset date of depression.

### 2.5 Criteria for use or nonuse of OHAs

In the present study, sulfonylureas, α‐glucosidase inhibitors, biguanides, thiazolidinediones, glinides, DPP‐4 inhibitors, and SGLT‐2 inhibitors were assessed. In order to assess these OHAs, which are used chronically, OHAs used on 80% or more of days within the duration of T2DM were included in statistical analysis. In addition, OHAs that had been used continuously for at least 30 days before the onset date of depression were also included (Figure 1C).

### 2.6 Statistical analysis

To compare differences in patient background between the case group and the control group, two‐tailed Student's *t*‐test or Wilcoxon rank‐sum test was performed for continuous data including age, duration of T2DM, and HbA_1c_. Chi‐squared test was performed for differences in categorical data including sex, hospital, medical history, and use of OHAs. P values were adjusted by false discovery rate (FDR) due to multiple comparisons.

The risk of depression with the different classes of OHAs was assessed by multiple logistic regression analysis; the presence or absence of depression was the dependent variable, the use or nonuse of OHAs was the independent variable, and covariables such as age, sex, HbA_1c_, duration of T2DM, hospital, and medical history were adjusted.

The level of significance was set to.05 for all statistical analyses. All statistical analyses were conducted with SAS software version 9.4 (SAS Institute Inc, Cary).

## 3 RESULTS

**Figure 2:**
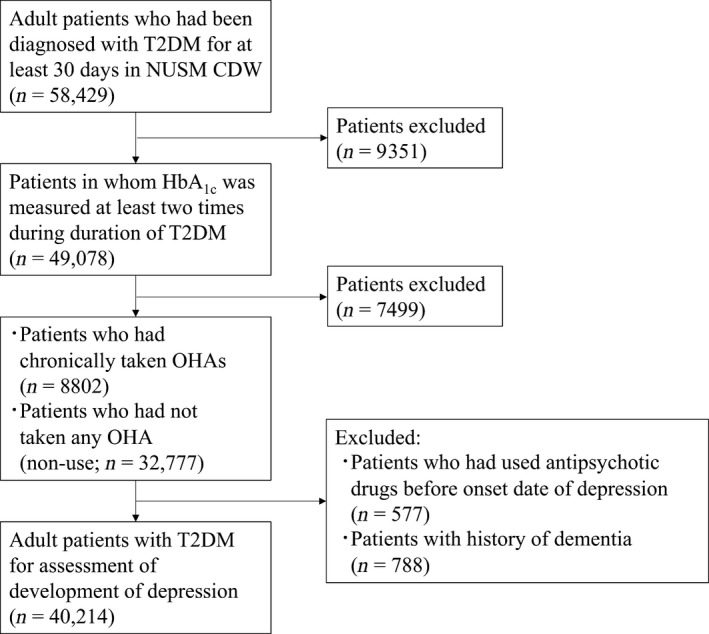
A flowchart of patient extractions from NUSM CDW

Figure 2 contains a flowchart depicting patient extractions from NUSM CDW. A total of 41 579 adult patients with T2DM were extracted from NUSM CDW. Of these patients, 1365 were excluded because of use of an antipsychotic drug or the development of dementia. Among the remaining 40 214 patients, 1979 [female: 1020 (50.54%)] who had developed depression were assigned to the case group, and 38 235 patients [female: 15 539 (40.64%)] who had not developed depression were assigned to the control group.

Characteristics of patients with T2DM are shown in Table 1. There was no significant difference in age between the case group (60.48 ± 13.71 years, mean ± standard deviation) and the control group (60.69 ± 13.36 years). On the other hand, sex, HbA_1c_, and duration of T2DM were significantly different between the two groups (*P* < .0001, respectively). The proportion of T2DM patients who developed depression showed no significant difference among the three hospitals (*P* = .0504). With regard to history of seven medical conditions, only kidney disease showed no significant difference between the two groups (*P* = .6278). However, statistical significance of the other six medical conditions (arrhythmia, hyperlipidemia, hypertension, rheumatoid arthritis, thyroid disease, and liver disease) was detected (*P* < .0001, respectively).

Characteristics of patients with T2DM related to the risk of depression are shown in Table 2. Age (adjusted odds ratio [AOR] for 10 years: 1.03; 95% confidence interval [CI]: 0.99‐1.07; *P* = .1211) 38 and duration of T2DM (AOR for 1 year: 1.00; 95% CI: 0.99‐1.01; *P* = .4089) 39 were not associated with the development of depression. However, the risk of depression in female patients with T2DM was significantly higher than that in male patients (AOR: 1.39; 95% CI: 1.26‐1.53; *P* < .0001). In addition, HbA_1c_ level was significantly associated with the development of depression (AOR for 1.0%: 1.18; 95% CI: 1.11‐1.26; *P* < .0001). There was no association between hospitals and the risk of depression. Regarding medical history, arrhythmia (AOR: 1.17; 95% CI: 1.06‐1.30; *P* < .01), hyperlipidemia (AOR: 1.23; 95% CI: 1.12‐1.36; *P* < .0001), hypertension (AOR: 1.34; 95% CI: 1.21‐1.49; *P* < .0001), rheumatoid arthritis (AOR: 1.22; 95% CI: 1.08‐1.37; *P* < .01), thyroid disease (AOR: 1.37; 95% CI: 1.24‐1.51; *P* < .0001), and liver disease (AOR: 1.34; 95% CI: 1.21‐1.48; *P* < .0001) were each significantly associated with the onset of depression. However, there was no association between kidney disease and depression (AOR: 0.97; 95% CI: 0.88‐1.08; *P* = .6045).

**Table 2:** Characteristics of patients with T2DM related to the risk of depression

Characteristics	Logistic regression coefficient	Adjusted		*P* value
		Odds ratio	95% CI	
Age, 10 years	.0282	1.03	0.99‐1.07	.1211
Female	.3286	1.39	1.26‐1.53	<.0001
HbA_1c_, 1.0%	.1678	1.18	1.11‐1.26	<.0001
Duration of T2DM, 1 year	−.0044	1.00	0.99‐1.01	.4089
Hospital (*vs* Surugadai)				
Itabashi	−.1071	0.91	0.82‐1.02	.1036
Hikarigaoka	−.0920	0.90	0.77‐1.04	.1597
Medical history				
Arrhythmia	.1601	1.17	1.06‐1.30	.0022
Hyperlipidemia	.2091	1.23	1.12‐1.36	<.0001
Hypertension	.2938	1.34	1.21‐1.49	<.0001
Rheumatoid arthritis	.1960	1.22	1.08‐1.37	.0014
Thyroid disease	.3159	1.37	1.24‐1.51	<.0001
Liver disease	.2912	1.34	1.21‐1.48	<.0001
Kidney disease	−.0263	0.97	0.88‐1.08	.6045
Oral hypoglycemic agents (*vs* nonuse)				
Sulfonylureas	.0386	1.04	0.83‐1.30	.7333
α‐glucosidase inhibitors	.0466	1.05	0.82‐1.34	.7081
DPP‐4 inhibitors	−1.1572	0.31	0.24‐0.42	<.0001
Biguanides	−.3160	0.73	0.52‐1.02	.0621
Thiazolidinediones	−.2847	0.75	0.51‐1.10	.1454
Glinides	−.2932	0.75	0.48‐1.17	.1974
SGLT‐2 inhibitors	−2.4260	0.09	0.01‐0.63	.0153

Note

Abbreviations: CI, confidence interval; DPP‐4, dipeptidyl peptidase‐4; SGLT‐2, sodium glucose cotransporter‐2; T2DM, type 2 diabetes mellitus.

After adjustment for patients’ characteristics including age, sex, HbA_1c_, duration of T2DM, hospital, and medical history, the use of DPP‐4 inhibitors was significantly associated with the risk of depression compared to nonuse of DPP‐4 inhibitors (AOR: 0.31; 95% CI: 0.24‐0.42; *P* < .0001). SGLT‐2 inhibitors also significantly decreased the risk of depression (AOR: 0.09; 95% CI: 0.01‐0.63; *P* = .0153), but the number of T2DM patients with depression who had taken SGLT‐2 inhibitors was very small (n = 1 in Table 1). The use of biguanides tended to decrease the risk of depression (AOR: 0.73; 95% CI: 0.52‐1.02; *P* = .0621). There was no association between the use of the other OHAs (sulfonylureas, α‐glucosidase inhibitors, thiazolidinediones, and glinides) and the risk of depression (*P* > .05, respectively).

## 4 DISCUSSION

As a result of this study, it is suggested that several characteristics including sex, high HbA_1c_, and medical history increase the risk of depression. Several reports that women have higher rates of depression compared with men regardless of the presence or absence of diabetes mellitus have been published.^40^, ^41^, ^42^ This finding that sex is a risk factor for depression has been supported by previous reports. Regarding glycemic control, it was suggested that high HbA_1c_ increases the risk of depression in the present study. Ravona‐Springer, et al have reported an association between HbA_1c_ variability and symptoms of depression.^37^ It is known that intensive glycemic control decreases the risk of diabetic complications including retinopathy, nephropathy, and neuropathy.^43^, ^44^ Therefore, glycemic control may play an important role in reducing the development of depression as well as preventing diabetic complications.

It is known that exercise in patients with T2DM not only improves peripheral insulin resistance but also lowers HbA_1c_ values.^45^, ^46^ In addition, exercise improves brain structural abnormalities and is associated with prevention of depression in depressed patients.^47^, ^48^ However, use of DPP‐4 inhibitors provides a lower risk of depression compared to nonuse of DPP‐4 inhibitors. Thus, the use of DPP‐4 inhibitors are more suitable than nonuse in terms of the risk of depression.

Brain insulin resistance is associated with depression.^29^, ^30^ It is known that thiazolidinediones (pioglitazone and rosiglitazone) and metformin reduce insulin resistance and improve insulin sensitivity.^6^, ^49^ In addition, these drugs penetrate the blood‐brain barrier (BBB).^50^, ^51^, ^52^ For this reason, these drugs may improve insulin resistance in brain tissue as well as peripheral tissue.^53^ However, the use of these drugs did not reduce the risk of depression in the present study. Therefore, it is suggested that improvement of insulin resistance in the brain does not decrease the risk of depression.

It was suggested that the use of DPP‐4 inhibitors and SGLT‐2 inhibitors decreases the risk of depression. However, it is unclear whether SGLT‐2 inhibitors decrease the risk of depression because the number of T2DM patients with depression who had taken SGLT‐2 inhibitors in this study was very small. DPP‐4 is responsible for the degradation of incretins such as glucagon‐like peptide‐1 (GLP‐1) and gastric inhibitory polypeptide. It has been suggested that GLP‐1 and its receptor agonists pass through the BBB.^54^, ^55^ GLP‐1 receptors are expressed in various regions of the brain including the hippocampus, neocortex, and cerebellum.^56^ Activation of the GLP‐1 receptor in the brain promotes neuroprotection in neurodegenerative disorders such as Alzheimer's disease, Parkinson's disease, and multiple sclerosis.^57^, ^58^, ^59^ An association between depression and neurodegeneration has been reported in several studies.^60^, ^61^ In addition, chronic activation of the GLP‐1 receptor in the brain of rats ameliorates depression‐like behavior.^62^ In this study, patients with T2DM had taken DPP‐4 inhibitors for at least 30 days. Therefore, chronic activation of GLP‐1 receptors in the brain may play a potential role in decreasing the risk of depression.

### 4.1 Limitations

The present study has some limitations. Firstly, some of female patients analyzed in this study have reached menopause. It is known that women who reached menopause have the second peak of schizophrenia.^63^ Therefore, menopause might have an effect on the risk of depression in female patients with T2DM.

Secondly, the number of patients who developed depression after the onset of T2DM was small irrespective of the class of OHAs. Especially, the use of SGLT‐2 inhibitors significantly decreased the risk of depression, but the number of patients using SGLT‐2 inhibitors was very small. Therefore, the risk of depression has not been assessed accurately in this study. GLP‐1 receptor agonists penetrate the BBB and activate GLP‐1 receptors in the brain.^54^, ^64^ However, no T2DM patients developed depression among those using a GLP‐1 receptor agonist in the present study. Thus, the effects of GLP‐1 receptor agonists on the risk of depression remain unclear.

Secondly, the present study was a retrospective, nonrandomized study with potential for selection bias and confounding factors. This study controlled potential confounding factors that were available and measurable, but failed to adjust for nonobserved confounding factors. Therefore, the findings obtained in this study should be verified through randomized cohort studies.

## DISCLOSURE

All authors declare no conflict of interest.

## AUTHOR CONTRIBUTIONS

HA and SA conceived of the presented idea. HA, KT, YN, TN, and YT developed the theory and performed data curation. HA, KT, YN, and YT verified the analytical methods and performed formal analysis. SA supervised the findings of this work. All authors discussed the results and contributed to the final manuscript.

## References

[1] GBD 2015 Disease and Injury Incidence and Prevalence Collaborators . Global, regional, and national incidence, prevalence, and years lived with disability for 310 diseases and injuries, 1990‐2015: a systematic analysis for the Global Burden of Disease Study 2015. Lancet. 2016;388:1545‐1602. 27733282 10.1016/S0140-6736(16)31678-6 PMC5055577

[2] World Health Organization . Fact sheet on depression.https://www.who.int/en/news-room/fact-sheets/detail/depression. Accessed May 9, 2019.

[3] Smith K . Mental health: a world of depression. Nature. 2014;515:181‐181. 25391942 10.1038/515180a

[4] van Dieren S , Beulens JW , van der Schouw YT , Grobbee DE , Neal B . The global burden of diabetes and its complications: an emerging pandemic. Eur J Cardiovasc Prev Rehabil. 2010;17:S3‐S8. 20489418 10.1097/01.hjr.0000368191.86614.5a

[5] Holden SH , Barnett AH , Peters JR , et al. The incidence of type 2 diabetes in the United Kingdom from 1991 to 2010. Diabetes Obes Metab. 2013;15:844‐852. 23675742 10.1111/dom.12123

[6] Garber AJ , Abrahamson MJ , Barzilay JI , et al. Consensus statement by the American Association of clinical endocrinologists and American College of endocrinology on the comprehensive type 2 diabetes management algorithm ‐ 2017 executive summary. Endocr Pract. 2017;23:207‐238. 28095040 10.4158/EP161682.CS

[7] Qaseem A , Barry MJ , Humphrey LL , Forciea MA . Clinical guidelines committee of the American College of Physicians. Oral pharmacologic treatment of type 2 diabetes mellitus: a clinical practice guideline update from the American College of Physicians. Ann Intern Med. 2017;166:279‐290. 28055075 10.7326/M16-1860

[8] Garber A , Henry R , Ratner R , et al.; LEAD‐3 (Mono) Study Group . Liraglutide versus glimepiride monotherapy for type 2 diabetes (LEAD‐3 Mono): a randomised, 52‐week, phase III, double‐blind, parallel‐treatment trial. Lancet. 2009;373:473‐481. 18819705 10.1016/S0140-6736(08)61246-5

[9] Raju A , Shetty S , Cai B , D'Souza AO . Hypoglycemia incidence rates and associated health care costs in patients with type 2 Diabetes mellitus treated with second‐line linagliptin or sulfonylurea after metformin monotherapy. J Manag Care Spec Pharm. 2016;22:483‐492. 27123911 10.18553/jmcp.2016.22.5.483 PMC10398174

[10] Maruthur NM , Tseng E , Hutfless S , et al. Diabetes medications as monotherapy or metformin‐based combination therapy for type 2 diabetes: a systematic review and meta‐analysis. Ann Intern Med. 2016;164:740‐751. 27088241 10.7326/M15-2650

[11] Umpierrez G , Tofé Povedano S , Pérez Manghi F , Shurzinske L , Pechtner V . Efficacy and safety of dulaglutide monotherapy versus metformin in type 2 diabetes in a randomized controlled trial (AWARD‐3). Diabetes Care. 2014;37:2168‐2176. 24842985 10.2337/dc13-2759

[12] Anderson RJ , Freedland KE , Clouse RE , Lustman PJ . The prevalence of comorbid depression in adults with diabetes: a meta‐analysis. Diabetes Care. 2001;24:1069‐1078. 11375373 10.2337/diacare.24.6.1069

[13] Rodríguez Calvín JL , Zapatero Gaviria A , Martín Ríos MD . Prevalence of depression in type 2 diabetes mellitus. Rev Clin Esp. 2015;215:156‐164. 25432144 10.1016/j.rce.2014.10.010

[14] Ali S , Stone MA , Peters JL , Davies MJ , Khunti K . The prevalence of co‐morbid depression in adults with type 2 diabetes: a systematic review and meta‐analysis. Diabet Med. 2006;23:1165‐1173. 17054590 10.1111/j.1464-5491.2006.01943.x

[15] Zhang Y , Ting RZW , Yang W , et al.; China Depression in Chinese Patients with Type 2 Diabetes (DD2) Study Group . Depression in Chinese patients with type 2 diabetes: associations with hyperglycemia, hypoglycemia, and poor treatment adherence. J Diabetes. 2015;7:800‐808. 25349949 10.1111/1753-0407.12238 PMC4964948

[16] Wang Y , Lopez JM , Bolge SC , Zhu VJ , Stang PE . Depression among people with type 2 diabetes mellitus, US National Health and Nutrition Examination Survey (NHANES), 2005‐2012. BMC Psychiatry. 2016;16:88. 27044315 10.1186/s12888-016-0800-2 PMC4820858

[17] Ali S , Davies MJ , Taub NA , Stone MA , Khunti K . Prevalence of diagnosed depression in South Asian and white European people with type 1 and type 2 diabetes mellitus in a UK secondary care population. Postgrad Med J. 2009;85:238‐243. 19520874 10.1136/pgmj.2008.074641

[18] Nouwen A , Winkley K , Twisk J , et al.; European Depression in Diabetes (EDID) Research Consortium . Type 2 diabetes mellitus as a risk factor for the onset of depression: a systematic review and meta‐analysis. Diabetologia. 2010;53:2480‐2486. 20711716 10.1007/s00125-010-1874-x PMC2974923

[19] Mezuk B , Eaton WW , Albrecht S , Golden SH . Depression and type 2 diabetes over the lifespan: a meta‐analysis. Diabetes Care. 2008;31:2383‐2390. 19033418 10.2337/dc08-0985 PMC2584200

[20] Lu FP , Lin KP , Kuo HK . Diabetes and the risk of multi‐system aging phenotypes: a systematic review and meta‐analysis. PLoS ONE. 2009;4:e4144. 19127292 10.1371/journal.pone.0004144 PMC2607544

[21] Jorm AF . Is depression a risk factor for dementia or cognitive decline? A review. Gerontology. 2000;46:219‐227. 10859462 10.1159/000022163

[22] Hirabayashi N , Hata J , Ohara T , et al. Association between diabetes and hippocampal atrophy in elderly Japanese: the Hisayama Study. Diabetes Care. 2016;39:1543‐1549. 27385328 10.2337/dc15-2800

[23] Katon W , Pedersen HS , Ribe AR , et al. Effect of depression and diabetes mellitus on the risk for dementia: a national population‐based cohort study. JAMA Psychiatry. 2015;72:612‐619. 25875310 10.1001/jamapsychiatry.2015.0082 PMC4666533

[24] Komsuoglu Celikyurt I , Mutlu O , Ulak G , et al. Exenatide treatment exerts anxiolytic‐ and antidepressant‐like effects and reverses neuropathy in a mouse model of type‐2 diabetes. Med Sci Monit Basic Res. 2014;20:112‐117. 25076419 10.12659/MSMBR.891168 PMC4138071

[25] Ostrovskaya RU , Yagubova SS , Gudasheva TA , Seredenin SB . Low‐molecular‐weight NGF mimetic corrects the cognitive deficit and depression‐like behavior in experimental diabetes. Acta Naturae. 2017;9:94‐102. PMC5509006 28740732

[26] Kim JM , Park CH , Park SK , et al. Ginsenoside Re Ameliorates brain insulin resistance and cognitive dysfunction in high fat diet‐induced C57BL/6 mice. J Agric Food Chem. 2017;65:2719‐2729. 28314104 10.1021/acs.jafc.7b00297

[27] Kang S , Kim CH , Jung H , Kim E , Song HT , Lee JE . Agmatine ameliorates type 2 diabetes induced‐Alzheimer's disease‐like alterations in high‐fat diet‐fed mice via reactivation of blunted insulin signalling. Neuropharmacology. 2017;113:467‐479. 27810390 10.1016/j.neuropharm.2016.10.029

[28] Kleinridders A , Cai W , Cappellucci L , et al. Insulin resistance in brain alters dopamine turnover and causes behavioral disorders. Proc Natl Acad Sci USA. 2015;112:3463‐3468. 25733901 10.1073/pnas.1500877112 PMC4371978

[29] Hamer JA , Testani D , Mansur RB , Lee Y , Subramaniapillai M , McIntyre RS . Brain insulin resistance: a treatment target for cognitive impairment and anhedonia in depression. Exp Neurol. 2019;315:1‐8. 30695707 10.1016/j.expneurol.2019.01.016

[30] Lyra E , Silva NM , Lam MP , et al. Insulin resistance as a shared pathogenic mechanism between depression and type 2 diabetes. Front Psychiatry. 2019;10:57. 30837902 10.3389/fpsyt.2019.00057 PMC6382695

[31] Kashiwagi A , Kasuga M , Araki E , et al.; Committee on the Standardization of Diabetes Mellitus‐Related Laboratory Testing of Japan Diabetes Society . International clinical harmonization of glycated hemoglobin in Japan: from Japan Diabetes Society to National Glycohemoglobin Standardization Program values. J Diabetes Invest. 2012;3:39‐40. 10.1111/j.2040-1124.2012.00207.x PMC4014931 24843544

[32] Koller EA , Doraiswamy PM . Olanzapine‐associated diabetes mellitus. Pharmacotherapy. 2002;22:841‐852. 12126218 10.1592/phco.22.11.841.33629

[33] Koller EA , Weber J , Doraiswamy PM , Schneider BS . A survey of reports of quetiapine‐associated hyperglycemia and diabetes mellitus. J Clin Psychiatry. 2004;65:857‐863. 15291665 10.4088/jcp.v65n0619

[34] Starkstein SE , Jorge R , Mizrahi R , Robinson RG . The construct of minor and major depression in Alzheimer's disease. Am J Psychiatry. 2005;162:2086‐2093. 16263848 10.1176/appi.ajp.162.11.2086

[35] Fuh JL , Wang SJ , Cummings JL . Neuropsychiatric profiles in patients with Alzheimer's disease and vascular dementia. J Neurol Neurosurg Psychiatry. 2005;76:1337‐1341. 16170072 10.1136/jnnp.2004.056408 PMC1739372

[36] American Diabetes Association . Classification and diagnosis of diabetes: standards of medical care in diabetes ‐ 2018. Diabetes Care. 2018;41(Suppl 1):S13‐S27. 29222373 10.2337/dc18-S002

[37] Ravona‐Springer R , Heymann A , Schmeidler J , et al. Hemoglobin A1c variability predicts symptoms of depression in elderly individuals with type 2 diabetes. Diabetes Care. 2017;40:1187‐1193. 28634202 10.2337/dc16-2754 PMC5864135

[38] Jeong JH , Um YH , Ko SH , et al.; Task Force Team for Diabetes Fact Sheet of the Korean Diabetes Association . Depression and mortality in people with type 2 diabetes mellitus, 2003 to 2013: a Nationwide Population‐Based Cohort Study. Diabetes Metab J. 2017;41:296‐302. 28868827 10.4093/dmj.2017.41.4.296 PMC5583407

[39] Rees G , Xie J , Fenwick EK , et al. Association between diabetes‐related eye complications and symptoms of anxiety and depression. JAMA Ophthalmol. 2016;134:1007‐1014. 27387297 10.1001/jamaophthalmol.2016.2213

[40] Collins MM , Corcoran P , Perry IJ . Anxiety and depression symptoms in patients with diabetes. Diabet Med. 2009;26:153‐161. 19236618 10.1111/j.1464-5491.2008.02648.x

[41] Ford DE , Erlinger TP . Depression and C‐reactive protein in US adults: data from the Third National Health and Nutrition Examination Survey. Arch Intern Med. 2004;164:1010‐1014. 15136311 10.1001/archinte.164.9.1010

[42] Breslau J , Gilman SE , Stein BD , Ruder T , Gmelin T , Miller E . Sex differences in recent first‐onset depression in an epidemiological sample of adolescents. Transl Psychiatry. 2017;7:e1139. 28556831 10.1038/tp.2017.105 PMC5534940

[43] Ismail‐Beigi F , Craven T , Banerji MA , et al.; ACCORD trial group . Effect of intensive treatment of hyperglycaemia on microvascular outcomes in type 2 diabetes: an analysis of the ACCORD randomised trial. Lancet. 2010;376:419‐430. 20594588 10.1016/S0140-6736(10)60576-4 PMC4123233

[44] ACCORD Study Group; ACCORD Eye Study Group , Chew EY , Ambrosius WT , et al. Effects of medical therapies on retinopathy progression in type 2 diabetes. N Engl J Med. 2010;363:233‐244. 20587587 10.1056/NEJMoa1001288 PMC4026164

[45] Motahari‐Tabari N , Ahmad Shirvani M , Shirzad‐e‐Ahoodashty M , Yousefi‐Abdolmaleki E , Teimourzadeh M . The effect of 8 weeks aerobic exercise on insulin resistance in type 2 diabetes: a randomized clinical trial. Glob J Health Sci. 2014;7:115‐121. 10.5539/gjhs.v7n1p115 PMC4796439 25560330

[46] Umpierre D , Ribeiro PA , Kramer CK , et al. Physical activity advice only or structured exercise training and association with HbA1c levels in type 2 diabetes: a systematic review and meta‐analysis. JAMA. 2011;305:1790‐1799. 21540423 10.1001/jama.2011.576

[47] Gujral S , Aizenstein H , Reynolds CF 3rd , Butters MA , Erickson KI . Exercise effects on depression: possible neural mechanisms. Gen Hosp Psychiatry. 2017;49:2‐10. 29122145 10.1016/j.genhosppsych.2017.04.012 PMC6437683

[48] Harvey SB , Øverland S , Hatch SL , Wessely S , Mykletun A , Hotopf M . Exercise and the prevention of depression: results of the HUNT Cohort Study. Am J Psychiatry. 2018;175:28‐36. 28969440 10.1176/appi.ajp.2017.16111223

[49] Zarifkar M , Noshad S , Shahriari M , et al. Inverse Association of peripheral orexin‐A with insulin resistance in type 2 diabetes mellitus: a randomized clinical trial. Rev Diabet Stud. 2017;14:301‐310. 29145540 10.1900/RDS.2017.14.301 PMC6115012

[50] Sheu WH , Chuang HC , Cheng SM , Lee MR , Chou CC , Cheng FC . Microdialysis combined blood sampling technique for the determination of rosiglitazone and glucose in brain and blood of gerbils subjected to cerebral ischemia. J Pharm Biomed Anal. 2011;54:759‐764. 21055895 10.1016/j.jpba.2010.10.008

[51] Grommes C , Karlo JC , Caprariello A , Blankenship D , Dechant A , Landreth GE . The PPARγ agonist pioglitazone crosses the blood‐brain barrier and reduces tumor growth in a human xenograft model. Cancer Chemother Pharmacol. 2013;71:929‐936. 23358645 10.1007/s00280-013-2084-2

[52] Łabuzek K , Suchy D , Gabryel B , Bielecka A , Liber S , Okopień B . Quantification of metformin by the HPLC method in brain regions, cerebrospinal fluid and plasma of rats treated with lipopolysaccharide. Pharmacol Rep. 2010;62:956‐965. 21098880 10.1016/s1734-1140(10)70357-1

[53] Hu SH , Jiang T , Yang SS , Yang Y . Pioglitazone ameliorates intracerebral insulin resistance and tau‐protein hyperphosphorylation in rats with type 2 diabetes. Exp Clin Endocrinol Diabetes. 2013;121:220‐224. 23512416 10.1055/s-0032-1333277

[54] Hunter K , Hölscher C . Drugs developed to treat diabetes, liraglutide and lixisenatide, cross the blood brain barrier and enhance neurogenesis. BMC Neurosci. 2012;13:33. 22443187 10.1186/1471-2202-13-33 PMC3352246

[55] Kastin AJ , Akerstrom V , Pan W . Interactions of glucagon‐like peptide‐1 (GLP‐1) with the blood‐brain barrier. J Mol Neurosci. 2002;18:7‐14. 11931352 10.1385/JMN:18:1-2:07

[56] Hamilton A , Hölscher C . Receptors for the incretin glucagon‐like peptide‐1 are expressed on neurons in the central nervous system. NeuroReport. 2009;20:1161‐1166. 19617854 10.1097/WNR.0b013e32832fbf14

[57] Salcedo I , Tweedie D , Li Y , Greig NH . Neuroprotective and neurotrophic actions of glucagon‐like peptide‐1: an emerging opportunity to treat neurodegenerative and cerebrovascular disorders. Br J Pharmacol. 2012;166:1586‐1599. 22519295 10.1111/j.1476-5381.2012.01971.x PMC3419902

[58] Duarte AI , Candeias E , Correia SC , et al. Crosstalk between diabetes and brain: glucagon‐like peptide‐1 mimetics as a promising therapy against neurodegeneration. Biochim Biophys Acta. 2013;1832:527‐541. 23314196 10.1016/j.bbadis.2013.01.008

[59] Lee CH , Jeon SJ , Cho KS , et al. Activation of glucagon‐like peptide‐1 receptor promotes neuroprotection in experimental autoimmune encephalomyelitis by reducing neuroinflammatory responses. Mol Neurobiol. 2018;55:3007‐3020. 28456941 10.1007/s12035-017-0550-2

[60] Manaye KF , Lei DL , Tizabi Y , Dávila‐García MI , Mouton PR , Kelly PH . Selective neuron loss in the paraventricular nucleus of hypothalamus in patients suffering from major depression and bipolar disorder. J Neuropathol Exp Neurol. 2005;64:224‐229. 15804054 10.1093/jnen/64.3.224

[61] Czéh B , Lucassen PJ . What causes the hippocampal volume decrease in depression? Are neurogenesis, glial changes and apoptosis implicated? Eur Arch Psychiatry Clin Neurosci. 2007;257:250‐260. 17401728 10.1007/s00406-007-0728-0

[62] Anderberg RH , Richard JE , Hansson C , Nissbrandt H , Bergquist F , Skibicka KP . GLP‐1 is both anxiogenic and antidepressant; divergent effects of acute and chronic GLP‐1 on emotionality. Psychoneuroendocrinology. 2016;65:54‐66. 26724568 10.1016/j.psyneuen.2015.11.021

[63] Häfner H . From onset and prodromal stage to a life‐long course of Schizophrenia and its symptom dimensions: how sex, age, and other risk factors influence incidence and course of illness. Psychiatry J. 2019;2019:9804836. 31139639 10.1155/2019/9804836 PMC6500669

[64] Kastin AJ , Akerstrom V . Entry of exendin‐4 into brain is rapid but may be limited at high doses. Int J Obes Relat Metab Disord. 2003;27:313‐318. 12629557 10.1038/sj.ijo.0802206

